# An example of the adaptation of the Nominal Group Technique (NGT) to a virtual format (vNGT) within healthcare research

**DOI:** 10.1186/s12874-024-02362-8

**Published:** 2024-10-15

**Authors:** Frances Riley-Bennett, Lal Russell, Rebecca Fisher

**Affiliations:** University of Nottingham, Nottingham, UK

**Keywords:** Nominal Group Technique, Methodology, Consensus, Virtual

## Abstract

The Nominal Group Technique (NGT) has been used to establish clinical priorities and generate guidelines within healthcare since its creation over fifty years ago. It is characterised by its five distinct stages; introduction, silent idea generation, ‘round robin’, clarifications and rating or ranking. A key element traditionally has been the inclusion of face-to-face discussion, however in the context of COVID-19 innovations were required. This article provides a case study illustrating an adaptation of the NGT to a virtual format (vNGT) and outlines the processes involved in a virtual NGT (vNGT), using an illustrative study exploring the rehabilitation of stroke survivors. The vNGT offers opportunities for global collaborations without the constraints of geography or incurred costs. Future studies should evaluate it’s acceptability for stroke survivors to enable their participation within research.

**Summary statement**

1. This study provides a guide for the use of virtual nominal group technique (vNGT), using a freely available video-conferencing platform

2. vNGT increases opportunities for global collaborations whilst incurring minimal costs.

3. It remains unclear how feasible this procedure is with patient populations who have potentially less digital confidence and access.

This work was supported by NIHR ARC-East Midlands, Grant number NIHR200171.

**Supplementary Information:**

The online version contains supplementary material available at 10.1186/s12874-024-02362-8.

## Introduction

There are two dominant consensus methodologies used within research, the Nominal Group Technique (NGT) and the Delphi technique [1]. For this study the NGT was chosen in preference to the Delphi technique due to the technique’s focus upon idea generation and the shorter time-period for completion [1]. Virtual ways of delivering the NGT were pursued as a result of the pandemic and will be presented throughout this article.

**Fig. 1:**
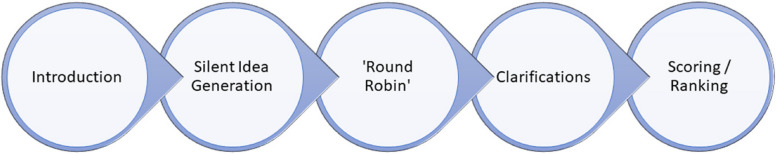
The five stages of the NGT

NGT is a structured consensus-generating activity developed within the field of social psychological research [1]. Since its creation, this technique has been widely used across many disciplines including industry, education and healthcare. Within healthcare it has been used to establish clinical priorities and generate guidelines [2–5]. The NGT process involves bringing an ‘expert’ panel together. ‘Expert’ status in this context refers to individuals who have sufficient knowledge about the discussed topic [6]. This panel then completes five distinct stages, see Fig. 1; following introductions, where the question(s) are posed for consideration, each member has time to silently generate ideas, these are then shared in a structured approach one at a time through a ‘round robin’ until the list is exhaustive [6]. This is followed by group discussion, not to sway agreement, but ensure ideas are clarified, promoting full understanding. Once this process is complete, each member individually scores or ranks the accumulated ideas, dependent upon the studies aim [6]. Ranking of ideas is associated with priority generation, whereas scoring the ideas creates consensus, this study uses the latter for consensus generation [7]. Potter et al., [8] emphasises that it is imperative that this protocol is adhered to ensure robust and reliable outputs.

Since 2010, there has been increased use of virtual research with advancements in online communication modalities [9], which have rapidly been accelerated by the global pandemic [10]. A fundamental component of the NGT process is group discussion, referring to face-to-face interactions, however in the context of COVID-19, meetings in this format were not permitted, necessitating an adaption of the methods [11]. There are examples where NGT have been completed “virtually” with the authors using different approaches [12, 13]. Kulczycki and Shewchuk (2008 (12)) utilised telephone conferencing and a web-based interface to establish strategies to improve the use of the female diaphragm and Tseng et al., [12] developed an NGT online platform for engineering education. The latter was a purely web-based platform with both synchronous (instant messaging) and asynchronous communication (discussion boards and ranking forms). A fundamental limitation of both studies was the skilled knowledge, resources and time required in order to build their technology platform. Jackson et al., [4] report using a virtual modified NGT to develop clinical guidelines for exercise in managing lower back pain. On closer scrutiny, their methodologically is closer to a modified Delphi method with rounds of questionnaires with the addition of a discussive element, therefore direct comparisons cannot be made [4]. The methods presented within this article use a freely available online video conferencing platform and were used to gain consensus regarding the core components of stroke community rehabilitation for survivors of stroke with severe disability as part of the HoRSSe study. This exemplar study will be used to illustrate the methods, full results are published elsewhere [5]. Ethical approval was granted by the University of Nottingham Faculty of Medicine and Health Sciences Research Ethics Committee, reference 27–0520, clinical trial number not applicable.

## Methods

The following case study [2], provides an in-depth description of the methods employed to adapt NGT used during the HoRSSe study from preparation through to analysis. This study utilized the online platform Microsoft Teams [14] however, other platforms are available with similar functionality. Microsoft Teams is a cloud-based communication platform which enables secure video meetings and screen sharing. All data was kept confidential and securely stored using Microsoft teams and the OneDrive according to protocols as recommended by the University of Nottingham [3].

Twelve participants were contacted through established clinical networks using guidance from Murphy et al., [15]. This aimed to provide breadth of representation across the country and professions. These were separated into two panels, each of six participants, following previous research utilising virtual focus groups [16]. In the exemplar study the following criteria were used to ensure optimal specialism and skills, conforming to the notion of ‘expert’: over 10-years' clinical or research experience within the severe stroke population and an understanding of the National Health Service (NHS). This study benefited from a wider collaborative research team comprising four specialists in stroke rehabilitation for the purpose of external scrutiny. For procedures regarding panel member composition and question formation the author directs the reader to Murphy et al., [15] who details this in-depth.

### PPIE

The Nottingham Stroke Research Partnership group, an established group of stroke survivors and their carers were involved in the design and conduct of this research. This is a voluntary group which works in partnership with the University of Nottingham. They have been instrumental in the agreement of adaptions as a result of the COVID-19 pandemic. This included contributing to the e-booklet and the vNGT questions. Two members of the group volunteered and participated within the idea generation stage of the nominal group which is discussed in further depth below.

## Preparation

In the preparatory stage, electronic versions of participant information and consent forms were sent via email to all proposed participants. All panel members provided written informed consent prior to participation. Following receipt of a signed electronic consent form, supporting information was provided as well as the electronic meeting link. For this study, the information was presented in an easily accessible e-booklet. A summary of the items contained in the e-booklet can be found in Table 1.

**Table 1:** Preparatory processes undertaken

Preparatory electronic booklet	
Summary of vNGT	A summary of the method as outlined in this article to ensure participants were aware of the process.
Questions for the vNGT	The vNGT questions were provided in advance so that participants could consider these prior to the meeting.
Context statement	The context statement was produced to set the scene from which participants were asked to base their ideas [15]. This included a definition of the study population, survivors of stroke with severe disability following discharge from inpatient rehabilitation. The setting was defined as home-based rehabilitation (including nursing and residential settings) within the UK NHS.
Literature review	A preliminary scoping review, unpublished, was provided summarising the evidence for survivors of stroke with severe disability. This ensured all participants were up to date on all relevant research and encouraged evidence-based idea generation [15].

Patient voice was included within this study. The e-booklet was provided to two PPIE members, a stroke survivor and carer of a stroke survivor. Both subsequently generated a list of ideas in response to the two questions. At their request they did not participate in the synchronous stages of the vNGT, citing fatigue and caring responsibilities respectively, instead their ideas were then presented during the round robin. This will be discussed in greater detail in stage 3.

The following vNGT protocol is based on Potter et al.’s [8] 5-stage adaption of the original Delbecq et al., [1], previously detailed above (Fig. 1). The five stages are detailed below and despite the necessary adaptation to a virtual platform, every effort was made to adhere to the original methodology protocol [8, 15]. A comparison between the original NGT and vNGT is tabulated below (Table 2).

**Table 2:** Comparison of stages of the standard NGT and vNGT

	Original nominal group technique	Virtual nominal group technique
Introductions	Completed face to face	Completed virtually through an online video meeting
Silent idea generation	Completed individually	Completed individually
Round robin	Completed face to face by a facilitator writing ideas on a white board or flip chart, or participants writing their ideas on post-its and placing them on a board.	Completed virtually through an online video meeting. A transcriber writing the ideas on a virtual shared document.
Clarification	Completed face to face	Completed virtually through an online video meeting.
Individual scoring	Completed individually at the end of the meeting	Completed individually virtually using an online voting platform within 72 h of the meeting.

### Stage 1—Introductions

All panel members and researchers signed on to the virtual meeting and introduced themselves (*n* = 6). Two researchers were present on the call, one to facilitate the vNGT (FRB) and the other to transcribe (LR). Using platform functionality, the session was recorded to enable researchers to return to the raw data, if required, at a later stage [14]. To start the formal process, the researcher provided an online presentation detailing the vNGT process, the context in which to base ideas and finally re-stating both questions for deliberation. This also provided an opportunity for any questions or clarifications. The questions used for HoRSSe study can be found in Table 3.

**Table 3:** Research questions used within the HoRSSe Study vNGT

*Question 1 “From an organisational perspective, what should a rehabilitation service for stroke survivors include in terms of structure and service delivery?” -*
*Question 2 “What interventions are needed to effectively rehabilitate this cohort of patients?”*

### Stage 2—Silent idea generation

To generate individual ideas in response to the questions, participants were asked to turn off their microphone and video function but remain connected to the online meeting. Approximately 15 min per question was allocated and a timer was displayed on the screen for all participants. During this time, facilitators (FRB and LR) were available via the platform’s chat function for support. For the exemplar study, potential domains were displayed alongside the timer, these were used to help organise their ideas. These were informed by literature and used as a deductive framework around which to organize discussion. A blank domain “other” was included to record new inductive insights offered by the panel [17] (see supplement 1). It was made clear that domains were used for organisation of ideas and not intended to restrict responses; participants were encouraged to respond freely. Whilst this is optional, using these domains helped with the later organisation of data. Following the first 15 min, the facilitator (FRB) checked to ensure the panel were ready to progress to the second question before resetting the 15-min timer whilst continuing to display the domains. Again, panel members remained muted with cameras switched off, and facilitators remained available for support via the chat function.

### Stage 3—Round Robin

Following the idea generation, video and microphone functions were re-instated. At this point the transcribing researcher (LR) shared their screen using the screen sharing function on the online platform [14]. This allowed a word document containing the domain table to be seen in real time by all participants (see supplement 1). Each participant, in turn, was invited to offer a single idea in response to question 1. This process continued until all ideas were exhausted, before progressing on to question 2. During this, the facilitator (FRB), in turn, presented the ideas previously generated by the PPIE members. All ideas were typed in real-time by the transcribing researcher (LR) onto the live word document, alongside the initials of the participant who proposed it. Transcription was non-verbatim with participants asked to confirm that the transcription accurately recorded their contribution. This document remained visible to participants throughout this stage.

### Stage 4—Clarification stage

To ensure a shared understanding amongst participants, panel members were asked to silently re-read the statements in a single domain. The facilitator asked each participant in turn if any statements in the domain required clarification. This provided an opportunity for discussion with the participant who presented the idea initially, identified by their initials if required. This process was completed domain by domain, which the authors would suggest for large data sets. The online meeting concluded once all participant members were satisfied and there were no outstanding queries.

### Stage 5—Individual scoring

Prior to sharing with the participants, the ideas generated were formatted into individual statements, without changing the manifest content. All statements were presented using an online platform (JISC) and distributed to participants who completed the entire vNGT process (*n* = 12) for scoring within 72 h of the online meeting [18]. This ensured the process did not lose momentum and the statements were clearly remembered by the participants. The manner in which NGT is scored is dependent upon it’s purpose; this exemplar was used to develop an unlimited amount of consensus statements, a 9-point numeric scale was used from not important / do not agree (1) to important /strongly agree (9) [19]. Other purposes include ascertaining priorities, see Thier and Mason [7] for alternative scoring protocols.

## Participant feedback

Case study requires researchers to draw data from multiple sources to gain a more robust understanding of the complex task under review [2]. Following completion of the vNGT, fully participantintg panel members (*n* = 12) were invited to provide feedback regarding the acceptability and useability of the vNGT process via email. These questions can be found in supplement 3. These included feedback regarding the five stages of the vNGT.

## Analysis

The HoRSSE study used two panels of six members that followed the above process. To compare and consolidate the statements and create overarching statements the following procedures were followed.

The voting responses from the statements online from both vNGTs were exported to Microsoft Excel to analyse for consensus. In concordance with standard NGT procedures, the level of agreement was set as 75% of all participants within the set ranges, 1 to 3, 4 to 6 and 7 to 9 not important, equivocal, and important, respectively [14]. In the case of strong disagreement, defined as one panel member voting 1 and another 9, outliers were removed, and the remainder reviewed for consensus [14]. The median and interquartile range for each individual statement and all statements which reached consensus were calculated to highlight dispersion around the consensus [14].

Analysis of NGT is commonly limited to consensus generation, however an optional addition of qualitative analysis can be useful to bring multiple panel groups together in order to compare and compile consensus [20].The HoRSSe study performed this optional stage, with the methods for combining multiple groups reported elsewhere [5, 20, 21].

Once finalized, the statements which achieved consensus, across both panels, were returned to the panel members for sense-checking.

## Findings

The illustrative HoRSSe study has been peer reviewed and published elsewhere, the findings are included in supplement 2 for reference [5].

## Participant feedback about the vNGT process

Nine participants (all health care professionals or clinical academics) provided feedback (*n* = 9, 75%). Overall, the respondents provided positive feedback for all aspects of the vNGT process, see Table 4.

**Table 4:** Results from participant feedback

Question	Median response
**2. Introduction questions …**	
2.1. The purpose was clear after the introduction	Strongly agree
2.2. The procedure was clear after the introduction	Strongly agree
2.3. The questions were clear after the introduction	Strongly agree
2.4. There was enough time to ask questions	Strongly agree
**3. Silent idea generation questions…**	
3.1. There was enough time during the silent idea generation	Strongly agree
3.2. The vNGT questions were clear during the silent idea generation	agree
3.3. Any questions were answered quickly and competently during the silent idea generation	Strongly agree / agree
**4. Round robin stage questions…**	
4.1. There was enough time to present all my ideas	Strongly agree
4.2. I felt comfortable to present my ideas	Strongly agree
4.3. I felt my voice was heard	Strongly agree
4.4. I was able to read the live round robin document sufficiently	Strongly agree
**5. Clarification stage questions…**	
5.1. There was enough time to clarify statements	Agree
5.2. I found the clarification stage beneficial	Agree
5.3. I felt able to ask questions to other panel members	Agree
**6. Voting stage questions …**	
6.1. I found the questionnaire easy to fill in	Agree
6.2. There was enough time to complete the questionnaire	Strongly Agree

## Discussion

This study illustrates the adaptation of the NGT into a virtual format. The benefit of this adaptation was the use of an established and freely accessible platform, Microsoft Teams, with no additional expertise or resources required [14]. Furthermore, literacy in the use of such platforms significantly increased due to necessity in 2020; the platform utilised being the chosen video conferencing platform for the NHS and UK universities throughout the pandemic [22]. Whilst this adapted methodology was a product of necessity, virtual adaptations could have far wider implications for use in a post-COVID landscape [11]. This includes logistical considerations such as accessing participants from wider geographical locations, places with limited meeting spaces or offering opportunities for wider national and international collaborations. The present study included experts from across the UK without incurring venue nor travel costs nor lost travelling time which makes efficient use of clinical time [23] When evaluating, participants acknowledged the efficiency and collaborative possibilities by using this virtual approach. Rupert et al., [24] suggest the potential for virtual environments in increasing the participation in research of seldom heard populations and those who are medically unwell.

The financial burden of vNGT is minimal in terms of physical resources; all documentation, correspondence and supporting information was provided online. The software was freely available as an app on mobile phones and computers. Whilst hardware such as mobile devices and laptops with web-cameras and access to broadband wi-fi are commonplace for most clinicians, this may still be a consideration if looking to utilise this methodology with other participants such as patient groups.

When evaluating their vNGT Tseng et al., [12] found high levels of satisfaction throughout the process from “conference procedures” to “being treated respectively” and “conference conclusion”. This is supported by the current study. The feedback provided by the participants was overwhelmingly positive. It is acknowledged that the survey respondents represent a self-selected sample, whilst a robust response rate (75%) was achieved the views of the minority who did not respond may have varied [25]. Kulczyzki and Schemchik [13], do not evaluate their methods from a methodological perspective, however they conclude their *“study validates the NGT as a means of collating expert opinion where little evidence exists” p229*.

A limitation of the vNGT was the occurrence of intermittent audio-feedback this is unfortunately common complication of virtual discussions [23]. Throughout the vNGT this was minimised by requesting participants mute when not contributing and the facilitator muting individuals if required.

There is the potential for virtual discussion to be inhibited when compared with face-to-face interactions, however Abrams et al., [26] found comparable levels of data richness from face to face and online focus groups. The structured and explicit nature of NGT offers frequent and repeated opportunities for participation. When asked for feedback, panel members in this study “strongly agreed” that they felt comfortable to present their ideas and that their voice was heard. To ensure synchronous functionality, typing responses on to the shared screen ensured all participants could see the ideas previously generated for reference. However, transcribing the participants ideas in real-time was challenging, and slowed down the process at times. In the future, researchers could explore two alternatives, firstly the use of live transcription, or secondly, in turn, each participant typing their idea into the chat section which could be pasted into the shared document by the transcribing facilitator.

Finally, the synchronous panel was solely composed of professionals, similar to that of Kulczyzki and Schemchik [13]. Whilst it is seen as a strength of the study that expert input from PPIE members was provided, they did not participate in the complete vNGT process nor subsequently the feedback stage. To help facilitate PPIE inclusion in future studies, consideration should be given to methods that support their inclusion even if this requires adaptation as presented within this study. This has been successfully completed in a face-to-face setting by Aspinal et al., [27], who completed 10 nominal groups regarding end-of-life preferences, each one grouped by homogeneous role, for example one solely a group of patients, another a group of relatives and another a group of district nurses. Similar to this study, their responses were then qualitatively analysed to generate comparisons and overall consensus. Whilst the two lay members involved with this study were digitally literate, it remains unclear how feasible the virtual procedure is with patient populations who have potentially less digital confidence and access [28]. Previous virtual focus groups have been successful amongst patients diagnosed with type 2 diabetes, however the authors noted they were unable to recruit participants over 60 years old to the virtual group, compared with 40% over 60 in the face-to-face group.

## Conclusion

To the authors knowledge this is the first study to outline the methods of a vNGT using a video conferencing platform. The future use of this method presents greater opportunities for global collaborations without the constraints of geography or associated travel costs. We have shown that it is an acceptable adaption of the original NGT, practical for health care professionals and clinical academics. The success of this study may have been dependent upon the technical literacy of the participants. Future studies should evaluate it’s acceptability for stroke survivors to enable their participation within research and therefore provide a perspective of those with lived experience.

## Supplementary Information

Supplementary Material 1.

Supplementary Material 2.

Supplementary Material 3.

## Data Availability

Data are available on reasonable request. Full content analysis categories available on request to lead author.

## Abbreviations

NGT: Nominal Group Technique
NHS: National Health Service
PPIE: Patient and Public Involvement and Engagement
vNGT: Virtual Nominal Group Technique
