# Supplementary material for: An example of the adaptation of the Nominal Group Technique (NGT) to a virtual format (vNGT) within healthcare research
Source: BMC Med Res Methodol. 2024 Oct 15;24:240. doi: 10.1186/s12874-024-02362-8 (PMC11476593; doi:10.1186/s12874-024-02362-8)
Supplement: Supplementary file 1 — Supplementary Material 1. [file 12874_2024_2362_MOESM1_ESM.docx]

Supplement 1

Domain tables used to collect and organise ideas generated by the panel.

**Question 1 domains**

| Team Composition |  |
| --- | --- |
| Time Frames |  |
| Outcome measures |  |
| Communication |  |
| Eligibility Criteria |  |
| Audit / Reporting |  |
| Other e.g. Collaboration with other services |  |

**Question 2 domains**

| Therapy |  |
| --- | --- |
| Pain / Spasticity |  |
| Home Environment / Family and Carer Support |  |
| Skin / Continence |  |
| Mood / Anxiety / Sleep / Relationships |  |
| Specialist seating / Wheelchairs |  |
| Other e.g. Palliative |  |
