# Supplementary material for: An example of the adaptation of the Nominal Group Technique (NGT) to a virtual format (vNGT) within healthcare research
Source: BMC Med Res Methodol. 2024 Oct 15;24:240. doi: 10.1186/s12874-024-02362-8 (PMC11476593; doi:10.1186/s12874-024-02362-8)
Supplement: Supplementary file 2 — Supplementary Material 2. [file 12874_2024_2362_MOESM2_ESM.docx]

# Supplement 2

Final consensus statements for the core components of home-based rehabilitation for survivors of stroke with severe disability

| **1.1 Core Multidisciplinary team** |
| --- |
| Multidisciplinary team (MDT) should consist of:   - Occupational Therapist - Physiotherapist - Psychologist - Nurse - Rehabilitation Support worker / Assistant Practitioner - Social care worker - Dietician - Speech and Language Therapist - Administrative support - Doctor |
| **1.2 Service eligibility criteria** |
| Individuals are eligible if they have:   - A diagnosis of stroke - A modified Rankin score of 4 or above - An identified stroke related need     Referrals into the service are not restricted:   - To healthcare professionals - By time since stroke - To patient's residence     Teams should have a broad focus, considering both restorative and compensatory approaches where appropriate to help patient and their carers:   - Participate in rehabilitation - Maximise their quality of life and participation in leisure activities - Manage their disability - Prevent secondary complications - Reduce carer burden |
| **1.3 Service Structure** |
| The MDT should:   - Form part of an integrated specialist stroke service that also delivers Early Supported Discharge (ESD). - Complete an initial holistic assessment within 1 week of discharge from hospital - Work in a coordinated manner, across disciplines and with established staffing ratios, in line with recognised ESD recommendations to provide rehabilitation over 7 days ([Link to guidance](https://www.ahajournals.org/doi/full/10.1161/STROKEAHA.110.606285)[23]) - Work flexibly in response to patient need including episodes of input focussing on achieving a specific patient goal, or facilitating carer involvement and self-practice, to reach the desired level of intensity. - Provide the opportunity for patients to receive 5 stroke specific sessions, 5 days a week, if deemed appropriate. - Provide length of input related to patient rehabilitation needs and goals; initial input available for minimum of 6 months, where appropriate, with the potential for re-referral if required. - Have strong leadership and weekly team meetings to review patient goals. - Support staff through the use of a debriefing system. |
| **1.4 Working across organisations** |
| Teams should:  In-reach into acute trusts to proactively support the discharge process and providing continuity of care, such as:   - Joint home visits - Involvement in discharge planning meetings - Making contact with the inpatient MDT, patients and carers prior to discharge, to support a seamless transition from inpatients to community.     Collaborate with:   - Care homes - GPs - Specialist teams such as tissue viability and palliative care services - Social care and continuing health - Community matron - Voluntary agencies e.g. Stroke Association     Support patient and carers to access:   - Exercise groups and gyms - Accessible transport - Life after stroke, patient and carer support |
| **1.5 Communication** |
| Contact should be made with patient, or if applicable with family or carers within 1 working day of discharge from acute hospital. Contact details for team members should be available for patients, families and carers.    Communication should be:   - Clear and transparent, in an accessible format for all patients, carers and healthcare professionals. - Coordinated and timely between the MDT, other agencies and services, patients and their carers (including care home staff). |
| **1.6 Audit** |
| Teams should participate in the Sentinel Stroke National Audit Programme (SSNAP) as well as other clinically relevant audit to inform clinical practice and regional commissioning of services.    Additional data captured could include:   - Unmet needs - 90-day mortality - Hospital readmissions (within 30 days) - Social care requirements |
| **1.7 Research** |
| Teams should be actively engaged in research, supporting patient enrolment and public and patient involvement throughout the process. Healthcare professionals should be leading research, disseminating findings and implementing them into clinical practice. This includes the sharing of case studies to demonstrate patient outcomes. |
| **2.1 Specific Interventions and MDT Skills** |
| The MDT should have the knowledge and expertise to anticipate need for this patient group and in managing and preventing secondary complications.    In addition to current national recommendations for the wider stroke population, the MDT should be able to offer the following specialist assessments and interventions specific to the needs of this patient group:   - Spasticity - Pain - Mobility transfers, including vehicle access - Postural support and seating - Skin integrity and continence - Environment including adaptation and equipment - Carer burden - Cognition including apraxia - Activities of daily living including basic self-care tasks - Communication, including access to computer software - Fatigue and sleep hygiene - Mood disorders - Sexual activity & relationships - Upper limb impairments - Medication management - Financial guidance - End of life care     The MDT should have sufficient training to recognise psychological problems (patient and carer), escalating when required, to ensure mood disorders are diagnosed appropriately.    If teams are unable to meet an identified patient or carer need, or more specialist expertise is required, they should access the appropriate service in a timely manner, such as:   - Video fluoroscopy - Spasticity management clinics - Pain management clinics - Specialist seating / wheelchair services - Orthotics - District Nurses - Stroke physician or GP - Dietician - Orthoptist / visual rehabilitation worker     Interventions should be:   - Based on the best available evidence. - Building in self-management alongside care~~-give~~r management. - Task specific and focussed on patient goals. The dose, structure and format of interventions should be specific to the individual patient to reach the desired level of intensity. - Facilitate carer and family involvement in rehabilitation to optimise opportunities for self-practice, where appropriate. - Designed to support and meet the needs of family and carers as well as patients |
| **2**.**2 Goal Setting** |
| Patients (and carers where appropriate) should be supported to engage in joint setting of goals with the MDT. Goals should be meaningful to the patient, aspirational and realistic, based on a holistic MDT assessment. Goals should link in with the foci of treatments detailed in section 1.2. |
| **2.3 Outcome Measures** |
| Validated outcome measures should be used, these should be sensitive to change in this population and specific to the individual’s impairment, participation and quality of life.  Wherever possible, the use of patient reported outcome measures (PROMs) and patient reported experience measures (PREMs) should be supported.    Outcome measures should be:   - Accessible for those with communication difficulties - Recorded within 2 weeks of discharge from hospital and reviewed at time points agreed with the patient and carer, appropriate to their individual needs. |
| **2.4 Education** **and training** |
| The team should be actively engaged in the education of:   - Commissioners and health care professionals regarding the rehabilitation needs and rehabilitation potential of this patient group. Raising awareness of meaningful outcomes for patients with severe disability following stroke, the impact of carer burden and the resources required. - Patients, families and carers regarding longer-term self-management*, secondary prevention and prevention of secondary complications such as shoulder pain. - Care Home staff, supporting ongoing rehabilitation across domains specific to the patient, including swallowing, positioning (including bed based), communication and moving and handling as well as enabling participation in leisure activities.     Providing practical training and written information, in an accessible format, for non-stroke specialist healthcare professionals, family and carers where appropriate.    * A process whereby individuals gain knowledge, skills and strategies to manage the physical, psychological, emotional and social effects of their long-term condition[24]. |
