# Supplementary material for: An example of the adaptation of the Nominal Group Technique (NGT) to a virtual format (vNGT) within healthcare research
Source: BMC Med Res Methodol. 2024 Oct 15;24:240. doi: 10.1186/s12874-024-02362-8 (PMC11476593; doi:10.1186/s12874-024-02362-8)
Supplement: Supplementary file 3 — Supplementary Material 3. [file 12874_2024_2362_MOESM3_ESM.docx]

Supplement 3

Virtual Nominal Group Evaluation questions

1.How would you describe your role?

Academic

Clinical academic

Clinician

2.Have you completed a nominal group before participating in the HoRSSe vNGT?

Yes

No

a.Was your previous experience virtual or face to face?

Virtual

Face to face

i.Please describe this previous experience in relation to the HoRSSe vNGT

3.Please rate the following... Scale poor (1) to good (10)

Please don't select more than 1 answer(s) per row.

|  | 1 | 2 | 3 | 4 | 5 | 6 | 7 | 8 | 9 | 10 |
| --- | --- | --- | --- | --- | --- | --- | --- | --- | --- | --- |
| Please rate the preparation documents provided prior to the vNGT |  |  |  |  |  |  |  |  |  |  |
| Please rate the presentations / information provided on the day of the vNGT |  |  |  |  |  |  |  |  |  |  |
| Please rate your understanding of the process following the information provided |  |  |  |  |  |  |  |  |  |  |
| Please rate the “facilitator” of the vNGT |  |  |  |  |  |  |  |  |  |  |

4.Introduction questions ...

Please don't select more than 1 answer(s) per row.

|  | Strongly Disagree | Disagree | Somewhat Disagree | Neither Agree nor Disagree | Somewhat Agree | Agree | Strongly Agree |
| --- | --- | --- | --- | --- | --- | --- | --- |
| The purpose was clear after the introduction |  |  |  |  |  |  |  |
| The procedure was clear after the introduction |  |  |  |  |  |  |  |
| The questions were clear after the introduction |  |  |  |  |  |  |  |
| There was enough time to ask questions |  |  |  |  |  |  |  |

a.Please write any comments regarding the introduction stage

5.Silent idea generation questions....

Please don't select more than 1 answer(s) per row.

|  | Strongly Disagree | Disagree | Somewhat Disagree | Neither Agree nor Disagree | Somewhat Agree | Agree | Strongly Agree |
| --- | --- | --- | --- | --- | --- | --- | --- |
| There was enough time during the silent idea generation |  |  |  |  |  |  |  |
| The vNGT questions were clear during the silent idea generation |  |  |  |  |  |  |  |
| Any questions were answered quickly and competently during the silent idea generation. |  |  |  |  |  |  |  |

a.Please write any comments regarding the silent idea generation stage

6.Round robin stage questions...

Please don't select more than 1 answer(s) per row.

|  | Strongly Disagree | Disagree | Somewhat Disagree | Neither Agree nor Disagree | Somewhat Agree | Agree | Strongly Agree |
| --- | --- | --- | --- | --- | --- | --- | --- |
| There was enough time to present all my ideas |  |  |  |  |  |  |  |
| I felt comfortable to present my ideas |  |  |  |  |  |  |  |
| I felt my voice was heard |  |  |  |  |  |  |  |
| I was able to read the live round robin document sufficiently |  |  |  |  |  |  |  |

a.Please write any comments regarding the round robin stage

7.Clarification stage questions...

Please don't select more than 1 answer(s) per row.

|  | Strongly Disagree | Disagree | Somewhat Disagree | Neither Agree nor Disagree | Somewhat Agree | Agree | Strongly Agree |
| --- | --- | --- | --- | --- | --- | --- | --- |
| There was enough time to clarify statements |  |  |  |  |  |  |  |
| I found the clarification stage beneficial |  |  |  |  |  |  |  |
| I felt able to ask questions to other panel members |  |  |  |  |  |  |  |

*a.*Please write any comments regarding the clarification stage

*8.*Voting stage questions ...

Please don't select more than 1 answer(s) per row.

|  | Strongly Disagree | Disagree | Somewhat Disagree | Neither Agree nor Disagree | Somewhat Agree | Agree | Strongly Agree |
| --- | --- | --- | --- | --- | --- | --- | --- |
| I found the questionnaire easy to fill in |  |  |  |  |  |  |  |
| There was enough time to complete the questionnaire |  |  |  |  |  |  |  |

a.Please write any comments regarding the voting stage

9.Experience: Scale poor (1) to good (10)

Please don't select more than 1 answer(s) per row.

|  | 1 | 2 | 3 | 4 | 5 | 6 | 7 | 8 | 9 | 10 |
| --- | --- | --- | --- | --- | --- | --- | --- | --- | --- | --- |
| Overall how would you rate your experience of the vNGT |  |  |  |  |  |  |  |  |  |  |

a.Would you participate in a vNGT again?

Yes

No

Maybe

i.Please explain you answer...

b.Please add any comments regarding the whole vNGT process
